# “Just not all ice users do that”: investigating perceptions and potential harms of Australia’s *Ice Destroys Lives* campaign in two studies

**DOI:** 10.1186/s12954-017-0175-9

**Published:** 2017-07-14

**Authors:** Caitlin H. Douglass, Elizabeth C. Early, Cassandra J. C. Wright, Anna Palmer, Peter Higgs, Brendan Quinn, Paul M. Dietze, Megan S. C. Lim

**Affiliations:** 1 Burnet Institute, 85 Commercial Road, Melbourne, Victoria 3004 Australia; 2 School of Public Health and Preventive Medicine, Monash University, 553 St Kilda Road, Melbourne, Victoria 3004 Australia; 3 Department of Public Health, La Trobe University, Plenty Road and Kingsbury Drive, Melbourne, Victoria 3086 Australia; 4 Melbourne School of Population and Global Health, University of Melbourne, Parkville, Victoria 3000 Australia; 5 Burnet Institute, 85 Commercial Road, Melbourne, Victoria 3004 Australia

**Keywords:** Injecting drug use, Australia, Media, Crystal methamphetamine, Young people

## Abstract

**Background:**

In 2015, the Australian government launched the media campaign *Ice Destroys Lives* targeting crystal methamphetamine use. Previous research indicates mass media campaigns may have harmful effects for people engaged in drug use. This study investigated perceptions and harms of *Ice Destroys Lives* among adults with a history of injecting drugs and young people.

**Methods:**

This analysis includes data from two studies: an online questionnaire with young people and in-depth interviews with adults who use crystal methamphetamine. Young people from Victoria, Australia, were recruited through Facebook. We collected data on drug use, campaign recognition and behaviours. Participants who recognised the campaign indicated whether they agreed with five statements related to *Ice Destroys Lives*. We compared campaign perceptions between young people who reported ever using crystal methamphetamine and those who did not. Adults who use crystal methamphetamine were sampled from the *Melbourne injecting drug user cohort study*. We asked participants if they recognised the campaign and whether it represented their experiences.

**Results:**

One thousand twenty-nine young people completed the questionnaire; 71% were female, 4% had used crystal methamphetamine and 69% recognised *Ice Destroys Lives*. Three quarters agreed the campaign made them *not want to use ice*. Ever using crystal methamphetamine was associated with disagreeing with three statements including *this campaign makes you not want to use ice* (adjusted odds ratio (AOR) = 4.3, confidence interval (CI) = 1.8–10.0), *this campaign accurately portrays the risks of ice use* (AOR = 3.2, CI = 1.4–7.6) and *this campaign makes you think that people who use ice are dangerous* (AOR = 6.6, CI = 2.2–19.8). We interviewed 14 people who used crystal methamphetamine; most were male, aged 29–39 years, and most recognised the campaign. Participants believed *Ice Destroys Lives* misrepresented their experiences and exaggerated “the nasty side” of drug use. Participants felt the campaign exacerbated negative labels and portrayed people who use crystal methamphetamine as “violent” and “crazy”.

**Conclusion:**

In our study, *Ice Destroys Lives* was widely recognised and delivered a prevention message to young people. However, for people with a history of crystal methamphetamine use, the campaign also reinforced negative stereotypes and did not encourage help seeking. Alternative evidence-based strategies are required to reduce crystal methamphetamine-related harms.

## Background

Australia has one of the highest rates of methamphetamine use worldwide [1]. In 2013, an estimated 7% of Australians aged 14 years and over reported using methamphetamines in their lifetime and 2% reported having done so in the past 12 months [2]. Currently, crystal methamphetamine (“ice”) is the purest and most commonly used form of the drug in Australia [2, 3]. There is evidence that methamphetamine-related harms (such as hospitalisations, mental health unit submissions for stimulant abuse and psychosis and arrests for possession or use) have increased in the state of Victoria since 2010 [4] in the context of major market changes [5]. Reports of increased harm have resulted in considerable community concern as evidenced by increased coverage of methamphetamine use in the Australian media [6]. In 2013, 16% of Australians aged 14 years and over rated methamphetamines as the drug of most concern in Australia compared to 40% in 2016 [7]. Illicit drug use is highly politicised in Australia with the majority of government funding spent on law enforcement (64%) and treatment (23%) compared to 10% on prevention [8].

In 2015, the Australian government launched a 6-week $9 million media campaign, *Ice Destroys Lives*, to target crystal methamphetamine use (referred to in the campaign as “ice”) [9, 10]. Mass media campaigns can disseminate drug-related information at a population level [11]. However, these campaigns are rarely evaluated, and most research has come from the USA [11–13]. An international systematic review investigating the effect of mass media campaigns on illicit drug use found that eight studies demonstrated no effect of media campaigns on drug use, four studies had beneficial effects and two studies reported increased drug use [12]. Generally, media campaigns target young people in an attempt to prevent new uptake; however, their widespread nature means that they also reach other populations such as people who already engage in drug use [14]. Anti-drug campaigns are often shock-based, reinforce negative stereotypes and portray people who use drugs as threats to the community [14]. Adverse implications for people who use drugs include isolation, low self-esteem and reduced access to treatment [14]. An evaluation of a US campaign—*The Montana Meth Project*—suggested that graphic advertisements had no effect on methamphetamine use among young people [15]. It was criticised for using sexualised, racialised and gendered advertisements that had the potential to influence policy, increase stigma and prevent the implementation of harm reduction strategies [16]. People with experience of methamphetamine dependency believed *The Montana Meth Project* was “demonising” and exacerbated their experiences of judgement, shame and rejection [14].

The tagline of Australia’s *Ice Destroys Lives* campaign was “Ice destroys lives. Don’t let it destroy yours”. The aims of the campaign were to contribute to “a reduction in the uptake of illicit drugs among young Australians” aged 14–25 years, to increase awareness of “a range of serious harms associated with the use of ice” among young people and their parents and “to encourage and support decisions not to use illicit drugs” [17]. The campaign used graphic advertisements to depict people who use crystal methamphetamine as violent, criminal and psychotic [18]. For example, one video advertisement featured a male character stealing from and physically assaulting his mother in the presence of a young child. The campaign was accompanied by a website which included educational information and links to a range of generalist support services such as Direct Line, Counselling Online and the Australian Drug Foundation [19].

The Department of Health commissioned an independent evaluation of the campaign, which suggested that *Ice Destroys Lives* increased both awareness of ice-related harms and negative attitudes towards ice among young people and their parents [17]. A total of 2171 young people were interviewed for this evaluation; one third thought that the campaign was relevant to them, and half indicated that they would “avoid using ice” as a result of seeing the campaign. However, the evaluation did not report whether participants had previously used crystal methamphetamine or other illicit drugs which may have influenced their opinion of the campaign. Importantly, the evaluation did not consider the effects of the campaign for other audiences such as people who already use crystal methamphetamine. In the existing literature, there is only a small number of studies describing the impact of anti-drug campaigns on people who use drugs [14]. Given the indication within the literature that these campaigns can be harmful, we analysed data from two ongoing studies (an online survey and a cohort study) to investigate potential harms of media campaigns targeting illicit drug use. The aims of this study were to:

1. Investigate recognition, perceptions and potential harms of *Ice Destroys Lives* among young people.
2. Explore recognition, perceptions and potential harms of *Ice Destroys Lives* among adults with a history of injecting drug use who report crystal methamphetamine use.

## Methods

### Design and setting

This study analysed data from two different studies with two different population groups. We used data from a large online questionnaire with young people that included a section of questions about the *Ice Destroys Lives* campaign. We also analysed interviews conducted as part of a cohort study with adults who had a history of injecting drug use who report crystal methamphetamine use to gather in-depth information.

### Online questionnaire with young people

The *Sex, Drugs and Rock ‘n’ Roll* study is an annual cross-sectional survey of young people conducted by the Burnet Institute. Methods have been described previously [20]. Briefly, we invited Victorians aged 15–29 years to complete an online questionnaire. Participants were recruited from social network sites through advertisements on Facebook and Instagram, and posts on Facebook pages that target young people (such as university pages and community youth groups). We collected data on participants’ socio-demographic characteristics, use of alcohol and other illicit drugs, and a range of other health domains. Upon completion, participants entered into a draw to win a $250 supermarket voucher. This study includes data from the 2016 questionnaire.

### Main outcome measures

We asked if participants recognised *Ice Destroys Lives* by providing five still images from the campaign. Those who recognised the campaign responded to five statements to determine their perceptions of the campaign’s accuracy and effectiveness. Participants stated whether they strongly agreed, agreed, neither agreed nor disagreed, disagreed or strongly disagreed with each statement. Levels of agreement were grouped in analysis in three categories: “agree” which combined “agree” and “strongly agree”, “neutral” (“neither agree nor disagree”), and “disagree” (“disagree” or “strongly disagree”). All statements were informed by drug experts and pilot-tested with young people.

### Analysis

All quantitative data were analysed using Stata version 13.1. Socio-demographic characteristics are presented as descriptive statistics. We used logistic regression to determine if any socio-demographic characteristics were associated with crystal methamphetamine use. We calculated the percentage of participants who agreed, disagreed or remained neutral with each statement on *Ice Destroys Lives*. We used multinomial logistic regression to calculate whether ever using crystal methamphetamine was correlated with agreeing, disagreeing or remaining neutral to statements about the campaign. We used “agree” as our reference category to produce odds ratios, *p* values and 95% confidence intervals. We ran a second multinomial logistic regression model that adjusted for socio-demographic characteristics that we identified as significantly associated with using crystal methamphetamine.

### Interviews with people who use crystal methamphetamine

The *Melbourne injecting drug user cohort study* (MIX) was established in 2008, the detailed methods for which have been described elsewhere [21]. Participants were initially recruited through street outreach and respondent-driven sampling. Participants complete an annual interview and provide in-depth information on drug use patterns, health service utilisation and other social outcomes. In 2016, MIX had 757 participants; the majority were male, the median age was 28 years, 59% had a history of incarceration and 6% reported that methamphetamine was the illicit drug they had used most in the past month [22]. Participants from MIX were eligible to complete an additional face to face in-depth interview focusing on their use of crystal methamphetamine if they (1) reported that they had used crystal methamphetamine more regularly than heroin or any other drug for a week or more since their baseline study visit, (2) could be contacted by telephone to arrange the interview and (3) provided written informed consent. Two trained interviewers (authors AP and PH) conducted these interviews, one section of which explored recall and perceptions of the *Ice Destroys Lives* campaign. Interviews ran between 30 and 60 min and participants were reimbursed $40 for their time and travel expenses. Interviews with MIX participants were audio-recorded and transcribed verbatim.

### Main outcome measures

Participants were asked if they recalled *Ice Destroys Lives*. Those who did not recall the campaign were shown an excerpt of the advertisement on the interviewer’s smartphone. All participants were asked open-ended questions about their perceptions of the campaign and whether it represented their experiences and use of crystal methamphetamine.

### Analysis

Interview transcripts were uploaded into NVivo 11 for processing and coding in a de-identifiable form. Authors CD and EE performed thematic analysis and used inductive, iterative coding. Transcripts were initially coded with broad open codes which were refined and converged during the process as deemed necessary. Researcher triangulation was also implemented, in which transcripts were read by the interviewers (AP and PH) to confirm the themes that arose from the analysis.

## Results

### Quantitative results from online questionnaire with young people

In 2016, we recruited 1029 young people. Their characteristics appear in Table 1. The majority were female, and the mean age was 23 years. In total, 54% of participants had ever used an illicit drug. Of all participants, 4% reported that they had used crystal methamphetamine in their lifetime and 1% had used it in the past month. Using crystal methamphetamine was significantly associated with being older, identifying as male, not living with parents, spending $120 or more per week for recreational purposes and identifying as non-heterosexual.

**Table 1:** Characteristics of participants by crystal methamphetamine use

	Total (*n* = 1029)	Had never used crystal methamphetamine in lifetime (*n* = 955)	Had used crystal methamphetamine in lifetime (*n* = 46)	Odds ratio	Confidence interval
	*n* (%)	*n* (%)	*n* (%)		
Gender					
Male	278 (27.0)	250 (26.2)	18 (39.2)	1.0	Ref
Female	731 (71.0)	688 (72.0)	25 (54.4)	0.5	0.3–0.9*
Non-binary, trans or other identity^a^	17 (1.7)	15 (1.6)	2 (4.4)	1.9	0.4–8.7
Born in Australia					
No	121 (11.8)	108 (11.3)	7 (15.2)	1.0	Ref
Yes	891 (86.6)	834 (87.3)	38 (82.6)	0.7	0.3–1.6
Completed post-high school education					
No	606 (58.9)	573 (60.0)	23 (50.0)	1.0	Ref
Yes	423 (41.1)	382 (40.0)	23 (50.0)	1.5	0.8–2.7
Live with parents					
No	498 (48.4)	446 (46.7)	36 (78.3)	1.0	Ref
Yes	531 (51.6)	509 (53.3)	10 (21.7)	0.2	0.1–0.5**
Money spent per week for recreation					
Less than $120	815 (79.2)	771 (80.7)	22 (47.8)	1.0	Ref
$120 or more	207 (20.1)	178 (18.6)	24 (52.2)	4.8	2.6–8.6**
Live in major city					
No	103 (10.0)	93 (9.7)	6 (13.0)	1.0	Ref
Yes	914 (88.8)	851 (89.1)	39 (84.8)	0.7	0.3–1.7
Heterosexual					
No	273 (26.5)	240 (25.1)	23 (50.0)	1.0	Ref
Yes	756 (73.5)	715 (74.9)	23 (50.0)	0.4	0.2–0.6**
Mean age (years)	22.5	22.4	25.2	1.3	1.2–1.5**

Percentages may not add up to 100% due to rounding. Numbers may not add up to total as participants who reported “I don’t wish to say” are not shown

*Ref* reference

*p < 0.05; **p < 0.001; ^a^Non-binary, trans and other identities have been combined due to small numbers

### Campaign recognition

The majority of participants recognised *Ice Destroys Lives* from the still images provided (69%). Among participants who recognised the campaign, 71% were female and the mean age was 23 years. Overall, 5% had ever used crystal methamphetamine (*n* = 33), and of these, 24% had used it in the past month (*n* = 8) (not shown in Table).

### Perceptions of *Ice Destroys Lives*

Participants’ perceptions of the *Ice Destroys Lives* campaign appear in Table 2. Of all participants, 75% agreed the campaign made them *not want to use ice*, 55% agreed it *accurately portrays the risk of ice use*, 46% agreed the campaign *will scare young people off using ice* and 84% agreed that the campaign made them think that *people who use ice are dangerous*. Overall, 47% disagreed that the campaign would *encourage ice users to seek help*.

**Table 2:** Young people’s perceptions of *Ice Destroys Lives* by crystal methamphetamine use

Statement	Total (*n* = 711)	Had never used crystal methamphetamine in lifetime (*n* = 678)	Had used crystal methamphetamine in lifetime (*n* = 33)	Odds ratio	95% confidence interval	Adjusted odds ratio^a^	95% confidence interval
This campaign	*n* (%)	*n* (%)	*n* (%)				
Makes you not want to use ice							
Agree	535 (75.4)	519 (76.7)	16 (48.5)	1.0	Ref	1.0	Ref
Neutral	77 (10.9)	73 (10.8)	4 (12.1)	1.8	0.6–5.5	1.0	0.3–3.3
Disagree	98 (13.8)	85 (12.6)	13 (39.4)	5.0	2.3–10.7**	4.3	1.8–10.0**
Accurately portrays the risks of ice use							
Agree	390 (55.3)	379 (56.4)	11 (33.3)	1.0	Ref	1.0	Ref
Neutral	209 (29.7)	205 (30.5)	4 (12.1)	0.7	0.2–2.1	0.5	0.2–1.7
Disagree	106 (15.0)	88 (13.1)	18 (54.6)	7.0	3.2–15.4**	3.2	1.4–7.6*
Makes you think that people who use ice are dangerous							
Agree	597 (84.1)	576 (85.1)	21 (63.6)	1.0	Ref	1.0	Ref
Neutral	86 (12.1)	81 (12.0)	5 (15.2)	1.7	0.6–4.6	1.0	0.4–3.1
Disagree	27 (3.8)	20 (3.0)	7 (21.2)	9.6	3.7–25.2**	6.6	2.2–19.8**
Will scare young people off using ice							
Agree	326 (46.0)	319 (47.2)	7 (21.2)	1.0	Ref	1.0	Ref
Neutral	211 (29.8)	201 (29.7)	10 (30.3)	2.3	0.8–6.1	1.5	0.5–4.2
Disagree	172 (24.3)	156 (23.1)	16 (48.5)	4.7	1.9–11.6*	2.6	1.0–6.9
Will encourage ice users to seek help							
Agree	146 (20.6)	142 (21.0)	4 (12.1)	1.0	Ref	1.0	Ref
Neutral	228 (32.1)	223 (32.9)	5 (15.2)	0.8	0.2–3.0	0.7	0.2–2.7
Disagree	336 (47.3)	312 (46.1)	24 (72.7)	2.7	0.9–8.0	1.8	0.6–5.5

Percentages may not add up to 100% due to rounding. Numbers may not add up to total as participants who reported “I don’t wish to say” are not shown

*Ref* reference

*p < 0.05; **p < 0.001

^a^Adjusted for gender, living arrangements, money spent per week for recreational purposes, sexual identity and age

### Perceptions of *Ice Destroys Lives* campaign by crystal methamphetamine use

Using crystal methamphetamine was associated with disagreeing with four of the five statements including *this campaign makes you not want to use ice* (OR = 5.0, CI = 2.3–10.7), *accurately portrays the risks of ice use* (OR = 7.0, CI = 3.2–15.4), *makes you think that people who use ice are dangerous* (OR = 9.6, CI = 3.7–25.2) and *this campaign will scare young people off using ice* (OR = 4.7, CI = 1.9–11.6) (Table 2). The exception was *this campaign will encourage ice users to seek help*; there was no significant association between disagreeing with this statement and reporting crystal methamphetamine use (OR = 2.7, CI = 0.9–8.0).

When we adjusted for age, gender, living arrangements, money spent per week and sexual identity, disagreeing with three of the five campaign statements remained significantly associated with reporting crystal methamphetamine use. However, the association between using crystal methamphetamine and disagreeing with the statement *this campaign will scare young people off using ice* did not remain significant (AOR = 2.6, CI = 1.0–6.9) (see Table 2).

### Qualitative results from in-depth interviews with adults who inject drugs

Sixty participants from MIX were eligible to participate in an in-depth interview focusing on crystal methamphetamine use. When researchers attempted to contact eligible participants, most phone numbers were disconnected. Of participants who could be contacted, 14 consented to participate and two declined. Most participants identified as male (*n* = 12) and were aged 29–39 years. Almost all participants interviewed were unemployed and lived in government housing. Approximately two thirds (*n* = 10) were on opiate substitution therapy. Nine of the 14 participants reported that heroin was their drug of choice at the time of the interview, while the remaining five nominated crystal methamphetamine as their drug of choice.

### Campaign recognition and recall

Recall of the *Ice Destroys Lives* campaign among adults who used crystal methamphetamine was mixed; six participants recalled seeing it, four reported they had not seen it and four participants were unsure. After being shown an excerpt of the video advertisement, those participants who were initially unsure reported that they did recognise the campaign.

### Perceptions of *Ice Destroys Lives* campaign

The main theme that emerged during the interviews was that *Ice Destroys Lives* misrepresented the experiences of people who use crystal methamphetamine. However, three participants acknowledged that some elements of the *Ice Destroys Lives* campaign were accurate, including sleep deprivation, hallucinations and the portrayal of crystal methamphetamine as a “very destructive drug”.

Yeah, that’s pretty accurate…I’ve been in some situations like that (male, 39 years).

Alternatively, nine participants felt that *Ice Destroys Lives* was sensationalised and exaggerated “the nasty side” of crystal methamphetamine use. Terms used to describe the campaign included “confronting”, “jumping the gun” and “pretty wrong”. When asked, five participants stated that the campaign was unlikely to prevent them from using crystal methamphetamine or encourage them to seek help, with one participant suggesting that “There just needs to be more help out there I think” (male, 35 years).

Despite acknowledging the harms of ice, most participants felt that *Ice Destroys Lives* did not accurately portray their personal experiences or behaviours. Their criticisms centred on the violence depicted in the campaign with seven participants stating that “it doesn’t apply to me”.

Like sometimes when I’m on it I get a bit rowdy but I don’t go out there causing trouble. I don’t want to get in trouble. I have my own things to do (male, 37 years).

While participants eagerly explained that the campaign misrepresented their personal circumstances and behaviours, they also believed that *Ice Destroys Lives* did not represent other people who use crystal methamphetamine. Ten participants mentioned that all people have different experiences when using the drug.

Just not all ice users do that. It’s basically saying that all ice users are likely to do that, you know (male, 38 years).

According to participants, *Ice Destroys Lives* depicted only the “worst case scenarios” to frame all people who use crystal methamphetamine in a negative light. Participants felt that *Ice Destroys Lives* exacerbated negative stereotypes and created fear throughout the community. They discussed the role of sensationalised media reporting contributing to assumptions and labels that community members imposed upon them.

Just some people think that you are going to go crazy or yeah. They just show so many people who have gotten violent whilst using ice and that, bashing bus drivers and that. They think that everyone who uses it is going to get like that…They think that all ice users will, just you know, bash them or rob them or do something (male, 38 years).

## Discussion

### Recognition and recall

This manuscript draws on data from two studies to explore perceptions of Australia’s *Ice Destroys Lives* campaign among young people and adults with a history of injecting drugs. Our results suggest that the majority of young people surveyed recognised the campaign, aligning with findings from the original evaluation of the campaign [17]. *Ice Destroys Lives* was also highly recognised by participants who used crystal methamphetamine, suggesting that it is important to consider the implications this campaign had on other audiences.

### Young people’s perceptions of *Ice Destroys Lives*

The main aim of *Ice Destroys Lives* was to “contribute to a reduction in the uptake of illicit drugs among young Australians”. In our questionnaire, three quarters of participants reported that the campaign made them *not want to use ice* and 46% agreed it would *scare young people off using ice*. This suggests that the campaign reached its target population with a prevention message, reflecting results from the original evaluation report [17]. However, our findings show that the campaign also had adverse effects: 84% reported that the campaign made them think that *people who use ice are dangerous* and 47% disagreed that the campaign encouraged help-seeking behaviours. This suggests that *Ice Destroys Lives* also delivered stigmatised messages to its target audience. There were significant differences in young people’s perceptions of *Ice Destroys Lives* based on crystal methamphetamine use. Participants who had previously used crystal methamphetamine were significantly less likely to believe that the campaign was accurate, helpful or effective. Alternatively, participants who had never used crystal methamphetamine were more likely to agree that the campaign made them *think that people who use ice are dangerous*. This reinforces the evidence that mass media campaigns such as *Ice Destroys Lives* contribute to negative stereotypes and are unlikely to increase help seeking for people who use crystal methamphetamine [12]. This provides support for allocating funds to targeted prevention and evidence-based strategies focusing on high-risk groups rather than generalist mass media campaigns targeting all young people [23].

### Perceptions of the campaign among adults who use crystal methamphetamine

When interviewed, adults with a history of injecting drug use who had used crystal methamphetamine generally did not identify with the violent behaviours depicted in *Ice Destroys Lives*. They distanced themselves from the deviant characters that were portrayed; however, they felt that the wider community still labelled them as “violent” and “crazy” based on the “worst case scenarios” shown. Participants felt that the campaign did not encourage them to seek help nor would it prevent them from using crystal methamphetamine in the future. Shock-based campaigns often frame crystal methamphetamine use as an individual-level problem [12, 14, 24] evidenced in this campaign by the tagline “Ice destroys lives. Don’t let it destroy yours”. Campaigns can also evoke fear throughout communities by linking crystal methamphetamine use to violence, criminal activity and immorality [25]. Language is important and can be stigmatising [26], and some suggest that use of aggressive and discriminatory language frames all people who use crystal methamphetamine as worthless and problematic [23]. This attitude can subsequently act as a barrier for treatment and increase stress and isolation among people who use crystal methamphetamine [26, 27]. Consumer representatives have encouraged the Australian government to alter their approach to ensure that future strategies are inclusive, evidence-based and cost-effective [23].

### Limitations

This study has some limitations. We recruited young people for the *Sex, Drugs and Rock ‘n’ Roll* survey through convenience sampling from Facebook and other social network sites. This sampling technique generally recruits a higher proportion of females than males, people with higher levels of education and individuals who have an interest in the topic [28, 29]; it is unlikely our sample is representative of all young Victorians. Only 33 participants from the online survey who were included in logistic regression had used crystal methamphetamine in their lifetime, and of these, 24% had used the drug in the past month. Consequently, confidence intervals lack precision, and results do not capture the perceptions of young people who have recently engaged in crystal methamphetamine use. Future studies should recruit a larger sample of young people engaged in drug use and differentiate results by usage patterns. Although statements in our survey were pilot-tested and informed by experts, we did not use a validated tool to measure young people’s perceptions of *Ice Destroys Lives*. In addition, although we adjusted for age, gender, living arrangements, money spent per week and sexual identity, there may have been other variables that influenced young people’s perceptions of *Ice Destroys Lives* such as political orientation and social networks [30]. All data were self-reported and may have been influenced by social desirability bias; however, this was minimised by the anonymity of the survey's online platform.

We recruited participants of the existing MIX study who use crystal methamphetamine through purposive sampling. Although this recruitment method does not capture a representative sample, it allowed us to access individuals who are typically hard-to-reach and could provide in-depth information for our research aims [21]. The participants we interviewed were a highly marginalised group; most were unemployed, lived in government housing and involved in treatment programs. It is likely that their past experiences increased their negative perceptions of the campaign. The MIX participants were significantly older than the target population of *Ice Destroys Lives* and had a history of injecting drugs; qualitative results are therefore not applicable to younger people who use crystal methamphetamine recreationally and may use by modes besides injecting. In future, it would be beneficial to evaluate similar campaigns with younger people based on patterns of usage. However, one aim of this study was to assess impacts of the campaign on those outside the target group; therefore, our study provides important information on the effects of media campaigns on adults with a history of injecting drugs. Initially, 60 MIX participants were eligible for interviews focusing on crystal methamphetamine; however, most of their telephone numbers were disconnected, and we were unable to contact them. Participants who were contactable by telephone may differ from the participants we were unable to reach. However, of participants who were contacted, the response rate was high and their socio-demographic characteristics reflected the MIX sample.

## Conclusions

*Ice Destroys Lives* was well recognised and delivered a prevention message to young people in our study; however, among young people who had ever used crystal methamphetamine, the campaign was less well perceived. Our study also provides important evidence that adults with a history of injecting drug use recalled this shock-based campaign but did not identify with the behaviours portrayed. Implications included feeling misrepresented and being negatively labelled, likely contributing further to discrimination and stigma within the community. The Australian government’s aim of targeting methamphetamine-related harms is commendable; funding for evidence-based strategies that are informed by experts and high-risk groups in an inclusive manner would be a more effective use of future resources.

## Abbreviations

MIX: Melbourne injecting drug user cohort study
